# Effect of pre-test rest duration on toe and ankle systolic blood pressure measurements

**DOI:** 10.1186/1757-1146-6-S1-P13

**Published:** 2013-05-31

**Authors:** Sean Sadler, Vivienne Chuter, Fiona Hawke

**Affiliations:** 1 Podiatry Department, The University of Newcastle, Ourimbah, NSW, 2258, Australia

## Background

Measurement of toe and ankle blood pressure is used to evaluate the peripheral arterial status of patients, yet the pre-test rest period is inconsistent in published studies and among practitioners, and could affect results. The aim of this systematic review was to evaluate all research that has investigated the effect of different periods of pre-test rest on toe and ankle systolic blood pressure.

## Methods

MEDLINE (from 1946), EMBASE (from 1947), CINAHL (from 1937), and Cochrane Central Register of Controlled Trials (CENTRAL) (from 1800) were searched up to April 2012. No language or publication restrictions were applied. Eighty-eight content experts and researchers in the field were contacted by email to assist in the identification of published, unpublished, and ongoing studies. Studies evaluating the effect of two or more pre-test rest durations on toe or ankle systolic blood pressure were eligible for inclusion. No restrictions were placed on participant characteristics or the method of blood pressure measurement. Outcomes included toe or ankle systolic blood pressure and adverse effects. Abstracts were independently assessed by two reviewers for potential inclusion.

## Results

1658 abstracts were identified by electronic searching. Thirty three of the 88 content experts and researchers in the field replied, identifying five potentially relevant studies. No studies were eligible for inclusion.

## Conclusion

There is no evidence of the effect of different periods of pre-test rest duration on toe and ankle systolic blood pressure measurements. Rigorous trials evaluating the effect of different durations of pre-test rest are required to direct clinical practice and research.

